# Published and Not Perished

**DOI:** 10.1371/journal.pbio.0030367

**Published:** 2005-10-11

**Authors:** Hemai Parthasarathy

## Abstract

*PLoS Biology* launches an electronic letters service.

Once upon a time, you formulated a hypothesis and designed the experiments to test it. You applied for a grant. You were awarded the money to pursue your line of inquiry. You did the work. You wrote the paper. Your colleagues reviewed your work and found it to be true. You published your paper. The conclusions of the paper joined the cannon of scientific knowledge. The End.

This platonic ideal of the scientific method is, sadly, at best science fiction and at worst history of science. The reality, as everyone knows, is much less linear—simultaneously more frustrating and more exciting. Publishing a paper is somewhere within an iterative loop that involves proving your point before you write the grant, working backwards to the rationale from a completely unexpected finding, and ultimately receiving a set of mixed peer reviews, which an editor interprets into a binary decision to publish your paper or not, in one peer-reviewed journal or another. And quite often, it is only with publication of the paper, that its worth is judged in earnest. Ultimately, worth is assessed by whether the scientific community decides to build upon a particular finding. More immediately, results are evaluated in journal clubs and scientific meetings, and a whole discussion surrounding a particular paper can be transmitted by word-of-mouth within a particular community, without ever being documented.

Last month, *PLoS Biology* launched its e-letters service, an electronic forum for responses to our published articles. Each article in *PLoS Biology* (including all content in the magazine section) could potentially have appended to it a series of comments that praise, criticize, clarify, or speculate about ideas presented in the original article.

Although open online discussion of content is quite common in some fields (e.g., medicine), and is all the rage in entirely different contexts (e.g., http://www.amazon.com), biology journals have been slow to embrace the discussion forum. Enthusiasm for the concept of attaching moderated blogs or discussion boards (e.g., http://www.slashdot.org) to research articles seems to emanate from the more junior members of the scientific community. Post-docs and graduate students seem especially sensitive to the wealth of discussion that surrounds published research articles that is never formally documented. Faced with an exponentially growing scientific literature, being “in the know” can provide a substantial advantage in pursuing promising leads and ignoring spurious tracks.

We hope that e-letters serve as the first step in implementing a broader program that provides a more interactive forum for publication of research. Although we have had requests to allow anonymous postings, we feel that there is enough anonymity in the publication process, and that transparent discussion—both positive and negative—should be encouraged. Ultimately, we would like to institute a system in which questions such as “How useful did you find this article?” and other sorts of immediate feedback mechanisms could serve as a tool to aid readers in allocating their limited time and attention.

Comments will be posted on *PLoS Biology* at the discretion of the editors. We will apply liberal policies to our screening process, but we will censor anything that is abusive, redundant, scientifically bogus, and/or extremely tangential to the issues addressed in the related article. As the *BMJ* noted on the anniversary of its 50,000th posting, there are downsides to a policy in which “just about anything that isn't libellous or doesn't breach confidentiality” is posted (BMJ 330: 1284). Those downsides included the following: “Some respondents feel the urge to opine on any given topic, and pile in early and often, despite having little of interest to say. Others have pet topics, which they return to obsessively, finding almost any peg to hang their views on. Some respondents don't seem to feel they're really alive until they've sparked off an angry response from someone else” (BMJ 330: 1284). [Fig pbio-0030367-g001]

**Figure pbio-0030367-g001:**
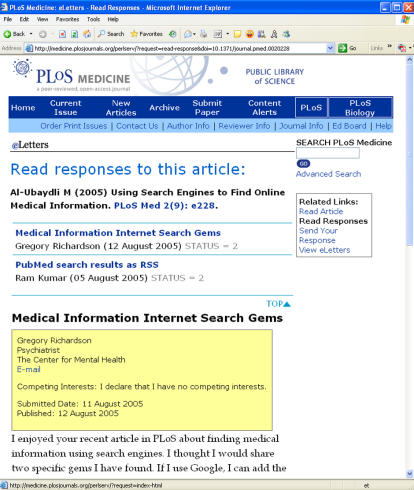
PLoS Medicine e-letters

Thus, we will endeavor to provide some level of oversight to foster a constructive dialogue about issues relevant to a particular article. Obviously, the degree of editorial oversight we can provide will, in part, depend on the volume of comments we receive. But we will cross that bridge when we get to it. Our sister journal, *PLoS Medicine*, launched e-letters last year, and at the present time, has posted 122 letters and rejected 45, a healthy but not unmanageable response.

We welcome your feedback on this new service, and we hope that you will use it to make open-access publishing a still more valuable resource within your scientific community. Write to us!

